# PROTOCOL: Psychosocial processes and intervention strategies behind islamist deradicalisation: A scoping review

**DOI:** 10.1002/cl2.1036

**Published:** 2019-09-05

**Authors:** Cátia de Carvalho, Isabel Rocha Pinto, Luís Filipe Azevedo, Alexandre Guerreiro, João Pedro Ramos, Mariana Reis Barbosa, Marta Pinto

**Affiliations:** ^1^ Centre of Psychology of University of Porto, Centre of Deviant Behaviour Sciences, Center for Health Technology and Services Research (CINTESIS) Faculty of Psychology and Education Sciences of University of Porto Porto Portugal; ^2^ Centre of Psychology of University of Porto Faculty of Psychology and Education Sciences of University of Porto Porto Portugal; ^3^ Department of Community Medicine, Information and Health Decision Science (MECIDS) & Center for Health Technology and Services Research (CINTESIS) & Cochrane Portugal Faculty of Medicine of University of Porto Porto Portugal; ^4^ School of Law University of Lisbon Lisbon Portugal; ^5^ Centre of Deviant Behaviour Sciences Fauclty of Psychology and Education Sciences of University of Porto Porto Portugal; ^6^ Center for Studies in Human Development, Faculty of Education and Psychology Catholic University of Portugal Porto Portugal; ^7^ Centre of Deviant Behaviour Sciences, Center for Health Technology and Services Research (CINTESIS) Faculty of Psychology and Education Sciences of University of Porto Porto Portugal

## 1 BACKGROUND

Since 1980, the world has witnessed an increase of Islamist terrorism attacks and these occurrences are proved to be the most lethal in comparison to other forms of terrorism (Piazza, 2009). Currently, this type of terrorism is also the most violent form of terrorism (Schmid, 2017) and it can be defined as terrorist activity perpetrated by terrorist groups that are inspired by radical and political interpretation of Islam, which involves spreading and imposing Islamic law through violence (Piazza, 2009; Schmid, 2017).

In a report written by Interpol in 2016, 15,000 foreign terrorist fighters (FTF) were deemed to be in Syria and in Iraq to join Islamist inspired terrorist groups, namely the Islamic State. In the beginning of the same year several countries in the European Union (EU) have reported a rising number of returning FTFs from Syria and Iraq due to the loss of Islamic State's (IS) occupied territory (Mehra, 2016; Reed, Pohl, & Jegerings, 2017; United Nations Security Council, 2016). A study commissioned by The Netherlands National Coordinator for Security and Counterterrorism (2016) found that 30% of FTFs who have left the EU are estimated to have returned and to be involved in planning, recruiting, or carrying out attacks. This clearly demonstrates the ability of terrorist organisations, such as IS, to mobilise returned FTFs and to involve homegrown extremists (Mehra, 2016). In addition, the wave of radicalisation rising across the globe and the effective dangers it poses to the world's security and stability is a clear sign of the urgency of counter‐radicalisation and deradicalisation measures (Kruglanski et al., 2014).

Generally, deradicalisation can be defined as the “methods and techniques used to undermine and reverse the completed radicalisation process, thereby reducing the potential risk to society from terrorism” (Clutterbuck, 2015). However, deradicalisation programmes are an under‐researched field of work (e.g., Bjorgo, 2011; Horgan & Braddock, 2010; Neumann, 2010), in need of exploration of their underlying principles, and scientific scrutiny about the main strategies and outcomes, so that they can be assessed, adapted and implemented in other countries.

Recently, a growing number of countries (viz. Egypt, Yemen, Saudi Arabia, Singapore, Indonesia, among others) have developed several deradicalisation programmes because of the concern regarding the release of convicted terrorists into society (Horgan & Braddock, 2010). These programmes aim to primarily change the radical behavior and to disengage people from terrorist organisations and violence (Demant & De Graaf, 2010; Drevon, 2015; Ganor & Falk, 2013; Gunaratna & Ali, 2009; Horgan & Braddock, 2010; Kropiunigg, 2013; Porges, 2010; Williams & Lindsey, 2014). For example, Yemen was the first country in the Middle East to develop deradicalisation efforts in its prisons (Porges, 2010). It started in 2002 and it aimed to change the ideological beliefs of terrorists through religious dialogue (Porges, 2010). In the case of Saudi Arabia, clerics, psychologists, and security officers work towards extremists’ rehabilitation, through education and training in order to reintegrate them into society (Kropiunigg, 2013; Porges, 2010; Williams & Lindsey, 2014). Another example comes from Egypt, where self‐deradicalisation occurred among Islamic militants in prisons (Drevon, 2015; Gunaratna & Ali, 2009). After this event, the efforts being implemented involve the process of persuading people to disengage from violence, through the creation of an environment that discourages the growth of extremism (Drevon, 2015; Gunaratna & Ali, 2009).

Regardless of all these programmes and strategies, there is no consensus on what constitutes success in a deradicalisation process (Horgan & Braddock, 2010; Porges, 2010) and this can only be achieved through a full consideration of the assessment of the differences among all programmes, taking into account their objectives, aims, targets, methods, and context (Clutterbuck, 2015). Similarly, there is no consensus on what triggers an individual to abandon terrorism (Horgan, 2009). Consequently, there is no available knowledge that may inform policy‐makers on how to critically think about what could be developed to facilitate or promote deradicalisation (Horgan, 2009).

Thus, this scoping review assesses studies related to Islamic deradicalisation and its main dynamics, programmes and strategies. In a context of uncertainty and lack of consensus, it is very important to map, gather, analyze and critically appraise knowledge produced on this topic in order to understand which are the main deradicalisation processes and practices, results achieved (positive or negative) and actors involved. This way, the results will inform policy‐makers and professionals working on this field about strategic decisions to approach the phenomenon, and identify gaps and future research needs.

The main objectives of this scoping review are to critically assess programmes being implemented to deradicalise Islamic extremists, to describe the contextual, economic and social factors underlying these programmes, and to describe the psychosocial characteristics of those being subjected to interventions. Thus, understanding these aspects will be valuable to inform policy‐makers and professionals working on this field, in order to develop and implement key strategies to deradicalise extremists and to contribute to counter‐radicalisation. Because this is a scoping review and not a systematic review, we do not specifically aim to assess the effectiveness of these programmes, but instead we will focus on critically and systematically mapping programmes being implemented and the psychosocial characteristics of those being subjected to interventions.

## 2 SPECIFIC OBJECTIVES

1. Produce practical knowledge about deradicalisation programmes;
  1.1. Describe the deradicalisation practices and programmes being implemented and their main characteristics.
  1.2. Describe the strategies and methods being used in deradicalisation programmes.
  1.3. Describe the psychosocial processes involved in deradicalisation.
  1.4. Describe the challenges associated with deradicalisation (namely, clarification of concepts and expectations, personnel and material resources constraints, lack of transparency).
2. Describe the contextual, social and economic factors being associated to deradicalisation in the literature.
3. Describe the psychosocial characteristics of the population involved in these programmes.

## 3 METHODOLOGY

### 3.1 Criteria for including and excluding studies

#### 3.1.1 Types of study designs

In order to capture the broadest scope of literature regarding Islamic deradicalisation programmes, we will include all types of studies under this topic. However, the general nature of the studies underlying deradicalisation programmes are theoretical and descriptive ones, not following empirical features and methodological considerations. This might be due to two factors: this field of study is under‐researched and, therefore, lacks comparative indicators; and the difficulty of accessing and following up participants subjected to deradicalisation programmes. If we manage to find primary studies with methodological features, we will address them as well. This way, we will also include opinion papers, reports, guidelines, systematic reviews, dissertations, conference proceedings, and other sources of information, as long as they target working proposals or already established Islamic deradicalisation programmes.

#### 3.1.2 Types of participants

Because this is a scoping review that aims to include as many studies as possible, the participants taken into consideration are the Islamic extremists that were subjected to any type of deradicalisation programmes deemed relevant. Typically, deradicalisation programmes target Muslim males, but in this study, we will address participants of any age, any country and both genders, as long as they have been involved with Islamic inspired terrorism and identify themselves as Muslims. This way, we will exclude studies that target deradicalisation programmes aimed at radicals with other background than Islamic‐inspired terrorism, for example, separatist terrorism.

#### 3.1.3 Types of interventions

As stated by Williams and Lindsey (2014), the deradicalisation interventions vary from country to country, are imposed by Governments and depend on the objectives of the programmes, as to whether they are aimed at changing behaviour or to change both behaviour and beliefs. These interventions are implemented in conjunction or separately by religious authorities, social workers, psychologists, and law enforcement personnel. Since this is a scoping review we will consider any type of intervention aiming at deradicalise and or disengage individuals from Islamic terrorism. Moreover, we will also consider any type of deradicalisation measures, even if it is not included in a deradicalisation programme, and also individual deradicalisation strategies.

#### 3.1.4 Types of outcomes

The principal outcome that we will look at in eligible studies will be the end of extremist violence and terrorist attacks. This is also the ultimate aim of implementing deradicalisation interventions, but in most cases, there are no sufficient follow‐up, or none at all, to know if this really happens. Another important outcome is the deradicalisation of beliefs. Although this is possible, this is something hard to assess and follow up. So, we will look for self‐reports and other records of deradicalisation statements. However, as this is a scoping review, a study not intended to pursue effectiveness assessment, we will not strictly focus on outcomes, since not every deradicalisation study presents results of their programmes.

#### 3.1.5 Types of settings

The most part of the deradicalisation programmes takes place in prisons (e.g., Ganor & Falk, 2013; Porges, 2010; Williams & Lindsey, 2014), nevertheless, we will include any type of setting, as long as it relates to deradicalisation concerning Islamic extremists.

### 3.2 Search strategy

In order to identify relevant studies, we established a comprehensive and broad search strategy combining published and unpublished literature. We do not plan to have geographical constraints, since we will consider literature from any country, and we will also include literature published until January 2018. Regarding language, we will include any study written in other language than English, by asking partners to help us with the review.

As recommended by Levac, Colquhoun, and O'Brien (2010), reviewers will meet at the beginning, midpoint and final stages of the process to discuss appropriateness and uncertainty of the study selection, to take decisions, and to refine the search strategy, taking into account the research question.

Following a strategy proposed by The Joanna Briggs Institute (2015) and by Arksey & O'Malley (2005), we will divide our search strategy into three stages:

1. Initial search: We conducted a limited search on two online databases relevant to the topic under consideration: Criminal Justice Abstracts and PsycINFO. After an analysis of the words in the titles and the abstracts of the relevant papers, we found that the relevant keywords are: deradicalisation, deradicalization, disengagement, counterterrorism, terrorism, rehabilitation, psychosocial, strategies, programmes, programmes. The search query will include three sets of keywords separated by AND Boolean operators corresponding to each of the three main search concepts (1‐deradicalisation, 2‐programmes/strategies, and 3‐counterterrorism) and within each concept keywords will be separated by OR Boolean operators.
2. Second search: A second search using all identified keywords will be conducted through important databases. Some of them are described as follows:

**Table:** 

Databases	
Campbell Library	United Nations Office of Counter‐Terrorism
Joanna Briggs Institute Library	United Nations Interregional Crime and Justice Institute
PsycINFO	Centre for the Study of Terrorism and Political Violence—University St. Andrews
PsycARTICLES	International Centre for Political Violence and Terrorism Research—Nanyang Technological University of Singapore
Criminal Justice Abstracts	Centre for Research on Extremism: The Extreme Right, Hate Crime and Political Violence
Psychology and Behavioral Sciences Collection	Quilliam Foundation
Criminal Justice Database—Proquest	German Institute on Radicalization and De‐Radicalization Studies
MEDLINE	RAN—Radicalisation Awareness Network
Academic Search Complete	International Institute for Counter‐Terrorism
Scopus	International Centre for the Study of Radicalisation and Political Violence
Web of Science Core Collection	Society for Terrorism Research
Current Contents Connect	Radicalisation Research
KCI—Korean Journal Database	SITE Intelligence Group
Open Dissertations	Centre de Prévention contre les Dérives Sectaires liées à l'Islam
Open Access Theses and Dissertations	Real Instituto Elcano
Proquest Dissertatiosn & Theses Open	Counter Extremism Project
Microsoft Academic	Brookings Institution
Theses Canada	Middle East Institute
Thèses France	START—National Consortium for the Study of Terrorism and Responses to Terrorism
Deutsche Nationalbibliothek	Hedayah—Countering Violent Extremism
BASE—Biefeld Academic Search Engine	Centre for Asymmetric Threat Studies
NARCIS	Danish Institute for International Studies
DiVA	RAND Corporation
The National Library of Wales	International Center for Counter‐Terrorism
RCAAP	NATO
Google Scholar	Europol
ResearchGate	Academia
3. Reference list:
  The process of searching and screening each study will be carefully reported and the details will be documented in a flow chart proposed by PRISMA (Liberati et al., 2009).

### 3.3 Description of methods used in primary research

The primary research that we have previously viewed have descriptive or theoretical features. This means that the studies found describe the main characteristics of the deradicalisation programmes, from the approaches and steps used in these interventions, the actors involved, to the expected or actual changes on radicals, without providing additional information about assessment, effectiveness and follow‐up (e.g., Drevon, 2015; Ganor & Falk, 2013; Williams & Lindsey, 2014). In other cases, primary studies only present deradicalisation programmes and elaborate some considerations about it, mainly about their characteristics and what could work (e.g., Veldhuis, 2012).

### 3.4 Details of study coding categories

The literature collected in eligible studies will be analysed in a meaningful manner to answer the research question, through a descriptive analytical method, proposed by Arksey and O'Malley (2005). To accomplish this method, the information will be analysed following Content Analysis (Bardin, 1977) and will be coded by two reviewers into the following categories:

General study features

- Author(s)
- Title
- Source/ Database
- Year of publication
- Peer‐reviewed
- Type of study
- Research methods

Deradicalisation programmes

- Country
- Objective/ pillars of the programme
- Name of the programme
- Promotor
- Methods/ strategies
- Psychosocial strategies
- Providers
- Setting
- Contextual factors
- Duration of programme
- Follow‐up

Participants

- Country
- Gender
- Age gap
- Background

– Family origin
– Religion
– Criminal activities
– Education
– Employment information

Outcome data

- Recidivism
- Number of participants in the programme
- Social integration
- Deradicalisation/ cognitive change
- Disengagement from violence/ behavioural change
- Challenges of the programmes
- Critics
- Other

In order to answer to the ultimate aim of performing this scoping review—inform policy‐makers and professionals working on this field about strategic decisions to approach the phenomenon, and identify gaps and future research needs—the results will be presented in Evidence Maps. This type of approach is defined as a systematic process to identify gaps in knowledge and/or future research needs in a broad field, and results are depicted in a user‐friendly format, such as graphs, figures or searchable databases (Miake‐Lye, Hempel, Shanman, & Sheklee, 2016). One of the main reasons authors chose to present results in Evidence Maps is that this approach shares some similarities with scoping reviews: the goals—review broad topics, and identify gaps/areas of future research‐, and the methodology used in the two approaches is the one proposed by Arksey & O'Malley (2005) (Miake‐Lye et al., 2016). However, there are three differences that will be tackled: Evidence Maps involve the consultation of an Advisory Board from the beginning of the search, promote a systematic search on online databases, and the results are shown in a visual depiction (Miake‐Lye et al. 2016). In this scoping review, the results will be presented in a cross‐tabular format and will be categorised in the following themes: deradicalisation interventions, setting, providers, methods, and outcomes.

### 3.5 Treatment of qualitative research

If we find qualitative studies, we will address them following the Content Analysis method proposed by Bardin (1977), which involves an objective, systematic, and quantitative description of manifested content in communications in order to interpret them. This means that eligible studies will be analysed thematically according to the scoping's objectives and coding categories, through immersion in the content and text dismemberment. If there is disagreement, another researcher will be consulted to decide the appropriateness of the content assigned to categories.

## ROLES AND RESPONSIBILITIES

- Content: Cátia de Carvalho, Mariana Reis Barbosa, Alexandre Guerreiro
- Systematic review methods: Marta Pinto, Luís Azevedo
- Statistical analysis: Luís Azevedo, Isabel Rocha Pinto
- Qualitative analysis: Cátia de Carvalho, Marta Pinto, Mariana Barbosa
- Information retrieval: Cátia de Carvalho, Marta Pinto, Luís Azevedo

## SOURCES OF SUPPORT

There are no sources of support to undertake this scoping review.

## DECLARATIONS OF INTEREST

Cátia de Carvalho and Mariana Reis Barbosa are involved in a project that aims to develop a training toolkit to desensitise youth from terrorist narratives. This is an ongoing project that ends by April 2018. Besides this is not a deradicalisation programme, the participation in this project does not interfere with the involvement in the scoping review. On the contrary, it might enrich it. It allows us to reach and contact relevant stakeholders, both from academia and civil society, in order to adequate and narrow our review to the real needs of society. Moreover, in this scoping review we have a strong and experienced team that ensures quality and exemption. In addition, we have no conflicts of interest related to the studies used in this scoping review.

## PRELIMINARY TIMEFRAME

- Search for literature: January 2018–February 2018
- Coding and assessment: March 2018–June 2018
- Initial results: June 2018
- Preparation of final paper: July 2018–October 2018
- Submission of final review: November 2018

## PLANS FOR UPDATING THE REVIEW

We intent to update our scoping review every 5 years.

## AUTHOR DECLARATION

### Authors’ responsibilities

By completing this form, you accept responsibility for preparing, maintaining and updating the review in accordance with Campbell Collaboration policy. The Campbell Collaboration will provide as much support as possible to assist with the preparation of the review.

A draft review must be submitted to the relevant Coordinating Group within 2 years of protocol publication. If drafts are not submitted before the agreed deadlines, or if we are unable to contact you for an extended period, the relevant Coordinating Group has the right to deregister the title or transfer the title to alternative authors. The Coordinating Group also has the right to deregister or transfer the title if it does not meet the standards of the Coordinating Group and/or the Campbell Collaboration.

You accept responsibility for maintaining the review in light of new evidence, comments and criticisms, and other developments, and updating the review at least once every 5 years, or, if requested, transferring responsibility for maintaining the review to others as agreed with the Coordinating Group.

### Publication in the Campbell Library

The support of the Coordinating Group in preparing your review is conditional upon your agreement to publish the protocol, finished review, and subsequent updates in the Campbell Library. The Campbell Collaboration places no restrictions on publication of the findings of a Campbell systematic review in a more abbreviated form as a journal article either before or after the publication of the monograph version in Campbell Systematic Reviews. Some journals, however, have restrictions that preclude publication of findings that have been, or will be, reported elsewhere and authors considering publication in such a journal should be aware of possible conflict with publication of the monograph version in Campbell Systematic Reviews. Publication in a journal after publication or in press status in Campbell Systematic Reviews should acknowledge the Campbell version and include a citation to it. Note that systematic reviews published in Campbell Systematic Reviews and coregistered with the Cochrane Collaboration may have additional requirements or restrictions for copublication. Review authors accept responsibility for meeting any copublication requirements.
